# Bacteriotherapy in Breast Cancer

**DOI:** 10.3390/ijms20235880

**Published:** 2019-11-23

**Authors:** Atieh Yaghoubi, Majid Khazaei, Seyed Mahdi Hasanian, Amir Avan, William C. Cho, Saman Soleimanpour

**Affiliations:** 1 Antimicrobial Resistance Research Center, Bu-Ali Research Institute, Mashhad University of Medical Sciences, Mashhad 91387-35499, Iran; yaghoubia961@mums.ac.ir; 2 Department of Microbiology and Virology, Faculty of Medicine, Mashhad University of Medical Sciences, Mashhad 91387-35499, Iran; 3 Department of Physiology, Faculty of Medicine, Mashhad University of Medical Sciences, Mashhad 9138735499, Iran; Khazaeim@mums.ac.ir; 4 Department of Medical Biochemistry, Faculty of Medicine, Mashhad University of Medical, Sciences, Mashhad 91387-35499, Iran; Hasanianmehrm@mums.ac.ir; 5 Cancer Research Center, Mashhad University of Medical Sciences, Mashhad 91387-35499, Iran; avana@mums.ac.ir; 6 Department of Clinical Oncology, Queen Elizabeth Hospital, Kowloon, Hong Kong

**Keywords:** breast cancer, bacteriotherapy, bacteriocins, bacterial peptide, toxin

## Abstract

Breast cancer is the second most common cause of cancer-related mortality among women around the world. Conventional treatments in the fight against breast cancer, such as chemotherapy, are being challenged regarding their effectiveness. Thus, strategies for the treatment of breast cancer need to be continuously refined to achieve a better patient outcome. We know that a number of bacteria are pathogenic and some are even associated with tumor development, however, recent studies have demonstrated interesting results suggesting some bacteria may have potential for cancer therapy. Therefore, the therapeutic role of bacteria has aroused attention in medical and pharmaceutical studies. Furthermore, genetic engineering has been used in bacterial therapy and may led to greater efficacy with few side effects. Some genetically modified non-pathogenic bacterial species are more successful due to their selectivity for cancer cells but with low toxicity for normal cells. Some live, attenuated, or genetically modified bacterias are capable to multiply in tumors and inhibit their growth. This article aims to review the role of bacteria and their products including bacterial peptides, bacteriocins, and toxins for the treatment of breast cancer.

## 1. Introduction

Breast cancer is the second most common cause of cancer-related mortality among women around the world. It is one of the most frequently diagnosed cancers in women and it has a high incidence rate in all countries [1]. According to the last estimate from the World Health Organization (WHO), in 2018, there were over two million new cases and it is estimated that 627,000 women died of the disease, which is approximately 15% of all cancer-causing deaths among women. We know cancer is a complicated disease result of genetic, epigenetic and environmental factors. Thus, it needs a range of treatment modalities and management [2,3].

Traditional and widely used treatments for cancer, including surgery, radiotherapy, chemotherapy, and some modern treatments (hormone-based therapy, stem cell therapies, immunotherapy, and dendritic cell-based immunotherapy) all have their own limitations. As an example, chemotherapeutic agents have nonspecific toxicity toward normal body cells [4,5]. Moreover, chemotherapy, in some cases, can lead to the formation of multidrug resistance cells. Due to the complications of chemotherapy, scientists have focused on examining the potential of using bacteria and their products to make new cancer medicines with low toxicity or without any side effect on normal cells. Genetic engineering has been used in bacteria therapy and has led to greater efficacy with minimal side effects. Some of genetically modified non-pathogenic bacterial species are more successful due to their selectivity for cancer cells with low toxicity for normal cells [6]. Metastatic cells can alter host cells, inhibit the mechanisms of normal growth of cells, and increase the rate of development of cell clones that produce neoplasm [7]. Thus, the treatment of cancer includes inducing apoptosis and decreasing or inhibiting the proliferation of tumor cells. Several bacterial strains have been identified and have natural oncolytic potential to penetrate, invade, and colonize in breast tumor [8].

Although many bacteria are carcinogens and tumor promoters [9,10,11,12,13,14,15], some have shown great potential towards cancer therapy. Several species of bacteria have shown an impressive ability to invade and colonize solid tumors, which has often led to neoplasm growth retardation and tumor clearance [6]. Different strains of *Clostridia, Lactococcus, Bifidobacteria, Shigella, Vibrio, Listeria, Escherichia*, and *Salmonella* have been evaluated against cancer in animal models [6]. However, some of these bacteria, such as *Bifidobacterium longum* and *Clostridium novyi* strains, are capable of colonizing the hypoxic area of the tumor, and therefore destroy the tumor cells [16,17]. Some others such as the attenuated auxotrophic mutants of the *Salmonella typhimurium,* one of the most efficient antitumor bacteria, can invade and destroy many types of cancer cells in vitro and replicate in toxic and hypoxic tumor regions in vivo [18]. For the first time, a bone surgeon, William B. Coley, used bacteria as an anticancer agent, known as Coley’s toxins. He injected a mixture of two heat-inactivated bacteria, *Streptococcus pyogenes* and *Serattia marcescence,* into more than 1000 patients with malignancy. Tumor regression was observed in many patients and 30 cases were completely treated [19]. Despite these great results, over time, development of other therapeutic approaches, such as radiation therapy and chemotherapy, caused the use of Coley’s toxins to gradually disappear. However, recent immunological studies suggest that the general principles of Coley’s toxins are correct because some cancers are sensitive to the enhancement and improvement of the patient’s immune system [20,21].

In spite of the side effects, bacteria-mediated tumor therapy (BMTT) has been used to control cancer for a long time. To use BMTT, it is necessary to maintain the balance between their side effects, such as infection, and its therapeutic effects. Bacillus Calmette-Guerin (BCG) is an attenuated strain of *Mycobacterium bovis* and it is the only bacterial agent that has been approved by the FDA for the treatment of non-muscle invasive bladder cancer (NMIBC), since the late 1970s. BCG has been used as a standard and is the most effective treatment for high-risk NMIBC [22,23]. Although clinical application of cancer bacteriotherapy is not very useful and routine, this approach can be promising in the future. Recently, advanced genetic engineering has increased the ability to change bacterial strains, which can help the production of non-hazardous bacteria that can treat cancer.

Most bacteria produce antitumor effects by reducing the nutrients required for the metabolism of cancer cells [24]. Obligate or facultative anaerobic bacteria find favorable niches within the hypoxic and necrotic regions of the tumor. The systemic administration of bacteria can lead to their entry in the tumor tissue, proliferation, and the formation of a necrotic region by reducing oxygen and the nutrient supply. Thus, it causes the tumor cells in the center of the solid tumor to die from starvation and suffocation [24]. Additionally, the bacteria, along with several other mechanisms have the ability to fight cancer cells, the most important of which include: (i) enhancing human immunity, (ii) as a carrier for cancer therapeutic agents, (iii) releasing substances (iv) forming biofilms, and (v) invading and colonizing the solid tumor [6].

In this article, we review the studies using bacteriotherapy (alone or combined with other methods) for the treatment of breast cancer, and critically discuss the impact of bacterial products (including, a bacterial peptide, bacteriocins, and toxins) that have the potential for anti-breast cancer treatment and mechanisms of bacteria-cancer-cell interaction.

## 2. Bacteriotherapy and Enhancing of Human Immunity

Among the cancer treatments, one therapeutic approach, known as immunotherapy is based on enhancing the host immune system against cancer [25]. There are different strategies used as an inhibitor of immune cells including monoclonal antibodies against tumor antigens, immune checkpoint inhibitors, adoptive cell therapies (e.g., CAR-T cells), and cytokine administration. Some of these strategies are already used in clinical practice for hematological malignancies such as a monoclonal antibody (anti-CCR4 mAb, Mogamulizumab) and a chemokine receptor inhibitor (CXCR4 antagonist AMD3100) [26,27]. Immunotherapy alters the expression of chemokines receptors in malignancies dictating leukocyte recruitment and activation, angiogenesis, and proliferation in the tumor [28]. Immune checkpoints are known as an important and effective form of immunotherapy that targets cytotoxic T lymphocyte-associated molecule-4 (CTLA-4), programmed cell death receptor-1 (PD-1), and programmed cell death ligand-1 (PD-L1) [29]. Furthermore, the main aim of the immunotherapy approach is to target the microenvironment of the tumor using the Toll-like receptors (TLRs) agonists that relate to the innate immune activation [30]. Despite the acceptable results, immunotherapy has faced challenges and limitations. One of the main drawbacks of immunotherapy is the lack of efficacy, meaning this approach does not work for every cancer patient and has efficacy only for a few cancers type [31]. Another challenge is that it is difficult to recognize clinically significant biomarkers, known as neoantigens or tumor-specific antigens (TSAs) expressed by tumor cells very slowly. TSAs are produced by normal and tumor cells which result in a lack of specific toxicity and a lower chance of success [25,31]. Furthermore, more predictive biomarkers are needed because, currently, only a few predictive biomarkers for cancer immunotherapy have been validated [32]. Moreover, heterogeneity within the tumor is another barrier to the success of immunotherapy [32], as well as the development of cancer which are resistant is another reason for therapeutic failures [33]. Additionally, immunotherapy agents are very expensive [34].

Interestingly, host and bacterial (as either pathogen or normal flora) interaction can increase the immune system of the host in various pathways. Tumor cells produce multiple modified surface antigens, reducing immunologic tolerance as carcinogenesis improvements, and various tumor-specific antigens are expressed. Because of the immunosurveillance, the immune response can clear abnormal cells in a two-way direction and a continuous way between the innate and adaptive immunity. In this process, the most important cells are cytotoxic T lymphocytes (T CD8+), natural killer cells (NK) as the main part of the innate immunity, macrophages, dendritic cells (DC), and regulatory T cells (T-reg) FOXP3+. These cells can interact with tumor cells and inhibit their growth.

The only effective response for tumor clearance is with T cells. CD8+ T cells have vital roles in the suppression of tumors, and they can kill tumor cells with cytotoxic molecules, such as granzymes and perforin. CD8+ T cells produce IFNγ and increase the expression of MHC class I antigens by the tumor cells, thus, making them better targets for CD8+ T cells. In addition, tumor-infiltrating lymphocytes, in breast cancer, comprise CD4+ T cells specific for class II restricted tumor antigens.

Hence, these cells are also able to carry out anticancer activity by providing help to CD8+ T cells or directly recognizing endogenously processed proteins on the surface of cancers eventually followed by secretion of type 1 cytokines or direct tumor killing.

Anaerobic bacteria, such as *E. coli,* are potent for the clearance of some tumor cells through stimulation of the host immune system, including lymphocytes T cells, which significantly associates with the antitumor activity. In the induction phase of bacterial infection, the only responsible effectors for tumor clearance are CD8+ T cells, whereas during the memory phase the clearance involves CD8+ and CD4+T cells. In some previous studies, cytotoxic T cell CD8+ has been demonstrated as having a critical role in the clearance of the original tumor after bacterial infection and CD8+T was able to destroy the already established tumors [35,36].

The immune system can also kill cancer cells by activating the inflammatory pathway. The defective *Salmonella typhimurium* strain in ppGpp synthesis activates the inflammasome pathways, and therefore it has therapeutic efficacy. The immune response is induced by the ΔppGpp *S. typhimurium* strain by damaging the signals released from the cancer cells, and significantly increases inflammatory cytokine IL-1β, TNF-α, and IL-18 in tumors, which results in tumor growth suppression of the most important arms of the immune system against tumor tissue, the tumor necrosis factor (TNF-α), which has the potential to destroy the vascular endothelial cells and forms a large hemorrhaging area within the tumor [37,38]. In one study, it was demonstrated that systematic administration of *Salmonella enterica serovar Typhimurium* could induce hemorrhaging and increase the entry of bacteria into the solid tumor, which causes necrosis.

## 3. Anticancer Substances Released by Bacteria

Recently, different bacterial products including toxin, peptides, bacteriocins, spors, and enzymes have gained attention as promising agents for the treatment of cancer. Moreover, in cancer bacteriotherapy, bacteria have been used alone or combined with conventional methods. They have also been used as target delivery vehicles for genes, drugs, or other therapeutic agents which have shown positive results on the regression of tumors and inhibition of metastasis in all the cases listed above. The use of bacteria or their products in the treatment of cancer is discussed below.

### 3.1. Bacteriocins in Cancer Therapy

Bacteriocins are ribosomally synthesized by bacteria and are generally described as peptides or proteins that could inhibit or kill other related or nonrelated bacterial strains. There are several large categories of bacteriocin that are categorized in several ways. Bacteriocins produced by gram-negative bacteria are classified according to their size, including microcins (less than 20 kDa in size), colicins (20 to 90 kDa in size), and tailocins (high molecular weight bacteriocins). Bacteriocins derived from gram-positive bacteria are typically classified into four different groups. Class I bacteriocins are small (less than 5 kDa) thermostable peptides that are post-translationally modified, also known as lantibiotics. These contain unusual amino acids such as lanthionine (Lan), methyllanthionine (MeLan), dehydroalanine (Dha), dehydrobutyrine (Dhb), and D-alanine (D-Ala). Some class I members include nisin, lacticin, and mersacidin [39,40]. Class II bacteriocins are small (<10 kDa) thermostable bacteriocins, having an amphiphilic helical structure that helps them penetrate into the membrane of the target cell. This class is subdivided into different subclasses including class IIa, IIb, and IIc. Pediocin PA-1 and sakacin A are examples of subclass IIa, subclass IIb bacteriocins include lactacin F and lactococcin G, and Gassericin A, circularin A, and carnocyclin A are members of subclass IIc [9,41,42]. Class III bacteriocins are large (>30 kDa) heat-labile bacteriocins, which are subdivided into two subclasses including bacteriolysins (subclass IIIa) and nonlytic proteins (subclass IIIb). Subclass IIIa is able to lysis the cell via degradation of the cell wall, and lysostaphin is one of the examples of this group that hydrolyzes the cell walls of several Staphylococcus species. Subclass IIIb causes the death of target cells by disrupting the plasma membrane. Some members of this class include megacins (of *Bacillus megaterium*), klebicin (of *Klebsiella pneumonia*), helveticin I (of *L. helveticus*), and enterolysin (of *E. faecalis*). Class IV is characterized as complex proteins containing lipid or carbohydrate moieties [2,43]. The result of a recent study demonstrated that bacteriocins could be used in the food industry as a preservative to extend the shelf life of products, and are non-immunogenic and practical in infectious disease treatment, while having specific toxicities for the cancer cell and cancer therapy (Table 1).

#### 3.1.1. Bovicin HC5

Bovicin HC5 is a broad-spectrum lantibiotic with a molecular mass of approximately 2440 kDa that is secreted from *Streptococcus bovis* HC5. Bovicin HC5 is known to have anti-breast cancer cell line activities in vitro such as MCF-7 [44,45].

#### 3.1.2. Colicins

Colicins are a family generally characterized as antibacterial cytotoxins synthesized by *E. coli* and other *Enterobacteriaceae*. Colicins are proteins of high molecular mass ranging from 40 to 80 kDa and released into the environment during the SOS response. Colicins use outer membrane proteins to bind to the target cell, and their lethal effect depends on their contact with the plasma membrane of sensitive cells.

Colicins affect sensitive bacteria cells via specific receptors in their walls to mediate their specific antibacterial effects. These receptors include porins (such as OmpF, FepA, TolQ, TolR, TolA, TolB, and Pal) located in the outer membrane proteins that have a natural role in small metabolite transport. In addition to their inhibitory effect on a sensitive bacterial cell wall, they have inhibitory effects on wall-less (L-form) cells, which have only the plasma membrane on their surface. This effect demonstrates that colicins could also inhibit eukaryotic cells. When colicin is transferred into the cytoplasm, the cytotoxic domain of colicin can kill the target cell by different kinds of effects such as depolarization of the cytoplasmic membrane (colicins A, B, E1, Ia, Ib, K, L, N, U, 5, and 10), a nonspecific DNase activity (colicins E2, E7, E8, and E9), a highly specific RNase activity (colicins E3, E4, E6, E5, and D), or by inhibition of murein synthesis (colicins M and pesticin). Another study reported the cytotoxic effects of colicin members on human solid tumor cell lines, and also colicin E1 and A are cytotoxic, inhibiting the breast carcinoma cell line including MCF7, ZR75, BT549, BT474, MDA-MB-231, SKBR3, and T47D [44,46].

#### 3.1.3. Laterosporulin 10

Laterosporulin 10 (LS10) is a peptide of 5.6 kDa produced by a gram-positive bacterium. Brevibacillus sp. LS10 is a defensin-like peptide which has antibacterial activity against a wide range of gram-positive and gram-negative pathogens. The N-terminal sequencing of LS10 is thermostable, and therefore this peptide shows low similarity with the existing antimicrobial peptides. The anticancer activity of this bacteriocin was investigated on a variety human cancer cell lines in vivo. Lower doses of LS10 induce apoptosis in cancerous cells, while higher doses of this bacteriocin cause necrotic death in the cancer cell. In addition, LS10 shows the highest activity against the breast cancer line MCF-7 cells [47].

#### 3.1.4. Nisin A

Nisin A belongs to the class I bacteriocins and is a polycyclic antibacterial peptide containing 34 amino acids derived from *Lactococcus lactis* subsp. This peptide has a broad-spectrum inhibitory effect on gram-negative bacteria and is extensively used as a food preservative. Moreover, nisin is able to inhibit and prevent the growth of multiple cancer cell lines by changing the integrity of the cell membrane and forming pores, and therefore changes the potential of the membrane [49]. Nisin A inhibits and prevents the local tumor invasion and metastasis of human cell lines of breast adenocarcinoma such as MCF-7 [48].

### 3.2. Bacterial Peptides in Cancer Therapy

Some anti-breast cancer peptides with bacterial origin are described in this section. A special kind of peptide is called non-ribosomal peptide (NRP). They are secondary bioactive metabolites that are synthesized by a complex of the enzyme called non-ribosomal peptide synthetases (NRPSs), which are present in bacteria, cyanobacteria, and fungi. These peptides have specific features in their chemical structures including D-amino acids, N-terminally attached fatty acid chains, N- and C-methylated residues, N-formulated residues, heterocyclic elements, and glycosylated amino acids, as well as phosphorylated residues. These secondary bioactive metabolites show widespread activity, involving anticancer and antimicrobial activities (Table 2).

#### 3.2.1. Ohmyungsamycins A and B

New cyclic peptides, called Ohmyungsamycins A and B, are produced by Streptomyces sp., isolated from a volcanic island in the Republic of Korea. These cyclic peptides contain unusual amino acids in their structure, including N-methyl-4-methoxytrytophan, hydroxyphenylalanine, and N, N-dimethylvaline. Both peptides show the anticancer activity, which could inhibit the growth of diverse cancerous cell lines and they also have antibacterial effects. Furthermore, these cyclic peptides show selective anti-proliferative activity against tumor cells as compared with normal cells. Ohmyungsamycins show the anticancer activity against human breast cancer cell line MDAMB231 [50].

#### 3.2.2. Azurin

Azurin (14 kDa, 128 amino acids) is a copper-containing metalloprotein with redox activity that plays a role in the denitrification process that is produced by *Pseudomonas aeruginosa*. Azurin acts as an electron transfer shuttle in *Pseudomonas aeruginosa* and other bacteria. It penetrates into the tumor cells, enhances the intracellular levels, and increases the stability of p53 by inhibiting COP1-mediated ubiquitination and proteasomal degradation, and therefore “p53” induces cell cycle arrest at G2/M and inhibits the development of cancer [59,60]. The domain of Azurin that is probably responsible is p28 (50 to 70 amino acids of azurin), containing 28 amino acids. It has a molecular weight of 2.8 kDa. p28, can penetrate human endothelial cells, and inhibit the kinase activity of VEGFR-2 (vascular endothelial growth factor receptor 2) and bFGF (basic fibroblast growth factor) that induce migration, capillary tube formation, and neoangiogenesis [61]. Furthermore, Azurin decreases the hyperphosphorylation of FAK (Focal adhesion kinase) and Src non-receptor tyrosine kinases associated with P-cadherin overexpression. P-cadherin (Pcad) overexpression occurs in 30% of invasive breast carcinomas and is associated with poor patient prognosis [62]. As an anticancer agent, “p28” has finished Phase I clinical trial as an investigational new drug application (IND 77,754) approved by the Food and Drug Administration [63]. Azurin has anticancer activity against various cell lines of breast cancer such as MCF7, ZR-75-1, T47D, MDA-MB-157, MDD2, and MDA-MB-231 [51,52]. Furthermore, different studies have demonstrated the anticancer activity of p28 on breast cancer models. The result of one study reported that p28 was able to significantly reduced the tumor size of MCF-7 xenografts in athymic mice after exposure to 10 mg/kg (3.4 μmol/kg) of peptide over the course of a daily dose for 30 days i.p [64]. Moreover, in another study, the MDA-MB-231 xenograft tumor was exposed to 10 mg/kg of p28 which resulted in inhibiting the tumor growth equal to or better than the IC50 dose of paclitaxel in MDA-MB-231 [65].

#### 3.2.3. Pep27anal2

Pep27anal2, (3.3–3.6 kDa, 27 amino acids) is an analogue of signal peptide Pep27, produced by *Streptococcus pneumoniae*. This peptide is able to initiate the cell death program in *S. pneumoniae*. It has also been found to have antimicrobial activity and inhibit the growth of cancer. Pep27anal2 is able to penetrate the cell membrane and induces caspase-independent and cytochrome-independent apoptosis. This peptide has anticancer activity in a variety of human cancer cell lines, and it is also able to reduce the proliferation of leukemia cells, gastric cancer cells, and additionally, it has an anticancer effect on the MCF-7 cell line of human breast cancer [53,54].

#### 3.2.4. Entap

Entap (6.2 kDa, 58–62 amino acids) is a newly discovered peptide with anticancer activity. Entap is an antiproliferative peptide produced by *Enterococcus* strains. The antiproliferative activity of Entap on human carcinoma cells has been examined by a study that demonstrated it inhibits the cell cycle at the phase of G1 and induces autophagous apoptosis. The antiproliferative activity of Entap has been examined in vitro on breast adenocarcinoma cell line MDA-MB-231 [43,56].

#### 3.2.5. Proximicins

Proximicins is a family of three novel aminofuran antibiotics produced by *Verrucosispora* strain. Proximicins B, with a molecular mass of approximately 413 kDa, is able to inhibit the growth of gram-positive bacteria, whereas proximicins C, with a molecular mass of approximately 436 kDa, has the inhibitory growth effect only on Brevibaccillus brevis. Surprisingly, gram-negative bacteria such as *Escherichia coli* K12, *Pseudomonas fluorescens*, *Proteus mirabilis*, and yeasts such as *Saccharomyces cerevisiae* are resistant to all of the proximicins. The unique features in the chemical structures of proximicins include 4-amino-furan-2-carboxylic acid, a hitherto unknown γ-amino acid. They are weak bactericidal peptides, but they show the powerful cytostatic activity on human breast carcinoma (MCF 7). An in vivo experiment of proximicin C on gastric adenocarcinoma (AGS) cells demonstrated that this peptide can provide cell cycle arrest at the G0/G1 phase after 24 h and increase the number of apoptotic cells after 40 h. It can also upregulate intracellular levels of p53 and the cyclin kinase inhibitor p21 in the AGS cells [57].

#### 3.2.6. Urukthapelstatin A

There is a new cyclic thiopeptide with the chemical formula C34H30N8O6S2, known as a Urukthapelstatin A (733kDa). This cyclic thiopeptide is isolated from Mechercharimyces asporophorigenens YM11-542 which is a marine bacterium. Recent data demonstrate that this thiopeptide has antitumor activity against human breast cancer cell lines MCF-7 [58].

### 3.3. Bacterial Toxins in Cancer Therapy

Toxins produced by the bacteria can destroy host cells and tissues and can also change the cellular processes, including control proliferation, apoptosis, and differentiation. Due to this toxin capability, some of them are tested for cancer treatment purposes (Table 3).

#### 3.3.1. Diphtheria Toxin

Diphtheria toxin (DT), with a molecular mass of approximately 60 kDa and 538 amino acids, is an exotoxin produced by *Corynebacterium diphtheria* [68]. Diphtheria toxin is produced due to the infection of bacteria with bacteriophage B. The gene that encodes diphtheria toxin is the “tox gene” that is present in some corynebacteriophages, therefore, the tox phage strains are able to secrete diphtheria toxin. DT is the combination of two subunits, “A” and “B”. Subunit “B” is responsible for binding to the receptor and translocation. Subunit “A” inhibits protein synthesis by ADP-ribosylation of cytoplasmic elongation factor 2 (EF-2), and therefore leads to cell death [75]. The anticancer activity has been shown in another study on experimental models and human’s diphtheria toxin (DT). The nontoxic mutant of diphtheria toxin is the cross-reacting material 197 (CRM197) that can bind to heparin-binding epidermal growth factor-like growth factor. CRM197 can inhibit the growth, reduce the angiogenesis, and induce the apoptosis in human adrenocortical carcinoma. Moreover, it acts as an immunological adjuvant and inhibits the heparin-binding epidermal growth factor [76]. DT is used in conjunction with other substances to reduce the related side effects. According to a study, the cytotoxicity activity of CRM197 is enhanced in combination with other substances. CRM197 in combination with doxorubicin, as the cytotoxicity of CRM197, was improved in a T-cell acute lymphoblastic leukemia cell lines. Additionally, the combination with cisplatin inhibits the growth and induces the apoptosis in glioma cells. DTAT is another substance with which the DT-based immunotoxin targets the tumor vascular endothelium. DTAT has anticancer activity in vitro against uPAR-expressing glioblastoma cells (U118MG, U373MG, and U87MG). Furthermore, it can be the cause of regression of small U118MG cell-induced tumors in mice. Diphtheria toxin has shown anticancer activity against breast carcinoma (MCF 7) [66,67,77].

#### 3.3.2. Botulinum Neurotoxin Type A

Botulinum neurotoxin type A (BoNT-A) is produced by strains of *Clostridium botulinum*, which can improve some kinds of cancer such as prostatic hyperplasia (BPH) via apoptotic activity, reducing the cell growth and proliferation. The molecular target of this neurotoxin is synaptic vesicle glycoprotein 2 (SV2) [78,79]. This protein has a major role in exocytosis and the secretory process in both synaptic and endocrine cells and tends to be overexpressed in cancer cells [80,81]. Furthermore, SV2 has been increasingly used, in recent years, as a molecular marker for several types of cancer. BoNT-A penetrates into the cancer cells through binding to a high-affinity SV2 receptor, which is exposed on the cell membrane during the exocytosis [82,83]. The BoNTA/SV2 complex is formed and influences the distribution of SV2 and the function of vesicles. This toxin could induce caspase-3 and -7 dependent apoptotic processes in breast cancer cell lines [80,84]. The inhibitory effect of Botulinum neurotoxin type “A” on SV2 expression has been determined on breast cancer cell lines including T47D, MDA-MB-231, and MDA-MB-453 [69,70].

#### 3.3.3. Exotoxin A

Exotoxin A is the 66 kDa peptide derived from *Pseudomonas aeruginosa*, which inhibits the synthesis of protein via ADP-ribosylation of elongation factor-2 (EF-2) [85]. This toxin is usually used as an immunotoxin with different ligands. *Pseudomonas* exotoxin A (PE), in conjugation with breast tumor selective antibodies (MAB), formed the immunotoxins with cytotoxicity effect on human breast tumor cell lines [72]. Another immunotoxin containing *Pseudomonas* exotoxin A, in conjugation with Herceptin antibody, can improve the efficiency of the Herceptin drug and improves the efficiency for treating breast cancer via overexpression of the HER2-neu receptor. Herceptin is a monoclonal antibody against Her2-neu receptor, which is overexpressed in some breast cancer cell lines and is used in targeted therapy [71]. Furthermore, this exotoxin induces the cytotoxicity and leads to death in cancer cell line by apoptosis. The anticancer effect of this toxin has been examined on different breast cancer cell lines including MCF-7, BT-20, CAMA-1, and SKBR-3 [71,72].

#### 3.3.4. Exotoxin T

Exotoxin T (ExoT) is produced by *Pseudomonas aeruginosa* that secretes via III secretion system (T3SS) and facilitates pathogenesis. ExoT contains two domains, i.e., an N-terminal with GTPase-activating (GAP) and a C-terminal that is a domain with ADP ribosyltransferase (ADPRT), and both domain activities contribute to ExoT-induced apoptosis [86]. Exotoxin T (ExoT) is different from *Pseudomonas* exotoxin A and diphtheria toxin that have a single putative target (e.g., eEF-2). This exotoxin has multiple cellular protein targets, which have critical roles in survival, proliferation, metastasis, and angiogenesis in cancer, by regulation of actin cytoskeletal dynamics, activation of protein kinases, cell cycle progression, cytokinesis, and the C10 regulator of kinases (CrkI, CrkII adaptor proteins) that are critical in the maintenance and formation of cellular focal adhesions and cytokinesis, and the glycolytic enzyme phosphoglycerate kinase 1 (PGK1) which is necessary for angiogenesis in cancer [87,88,89]. Thus, the resistance of cancer cells to cytotoxicity induced by ExoT is highly unlikely. In vitro studies have demonstrated that ExoT can cause cytotoxicity to reduce tumor establishment and growth in multiple cancer and breast cancer cell lines including MDA-MB-231, EMT6, 4T1 [73].

#### 3.3.5. Hyaluronidase (HylP)

The hyaluronidase (Hyals) is an enzyme from *Streptococcus pyogenes* which is able to degrade predominantly hyaluronan (HA), and it exhibits an anticancer activity on different cancers, as well as breast cancer. One of the features observed in many solid tumors is an increase in the extracellular matrix (ECM) deposition. Hyaluronan (HA) is one of the ECM components that increased in many solid tumors, leading to a decrease in the elasticity of tumor tissue and an increase of interstitial fluid pressure [90,91,92]. Recent studies have demonstrated that the addition of hyaluronidase to chemotherapeutic regimens could significantly improve efficacy [93,94]. In one study, Hs578T, MDA-MB-231, and MCF-7 cell lines were exposed to the bacteriophage H4489A hyaluronidase (HylP) of *Streptococcus pyogenes*. The result suggested that the inhibitory and invasive effect of HylP is due to a reduction of the Hyaluronan (HA) that is a nonsulfated glycosaminoglycan present in the extracellular matrix of tissue homeostasis and structural integrity [95].

## 4. Bacteria as a Carrier for Cancer Therapeutic Agents

### 4.1. Salmonella Typhimurium

*Salmonella typhimurium* is a pathogenical gram-negative bacteria found in the intestinal lumen. Toxicity activity of *Salmonella typhimurium* is due to an outer membrane feature of lipopolysaccharides (LPS), which protect it from the environment. Various studies have demonstrated that the attenuated strains of *Salmonella* can provide the anticancer activity [96] (Table 4).

**Figure 1:**
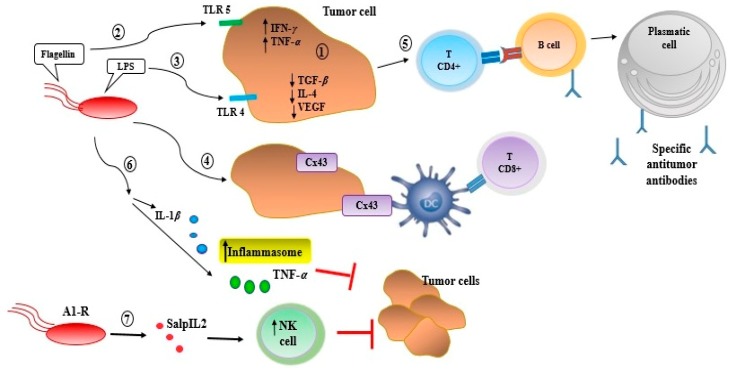
Activation of innate and adaptive immune response in the tumor microenviroment by *Salmonella*. Once *Salmonella* colonizes tumor tissue, it induces an antitumor innate and adaptive immune response through several mechanisms as follows: (i) It promotes proinflammatory cytokines (IFN-*γ* and TNF-*α*), while decreasing both anti-inflammatory (TGF-*β*, IL-4) and angiogenic factors (VEGF) associated with tumor growth progression; (ii and iii) interactions between bacterial components (LPS and flagellin) and tumor cell receptors as TLR4 or TLR5, respectively, induce a cytokine secretion that promotes the recruitment of neutrophils, macrophages, T lymphocytes, B lymphocytes, and dendritic cells to the tumor microenvironment; (iv) *Salmonella* colonization induces the expression of connexin 43 (Cx43), a molecule that plays a major role in the cross-presentation of tumor antigens by dendritic cells (DC) to CD8+ T-cells; (v) the presence of antitumor CD4+ T-cell induces the activation and differentiation of B lymphocytes in plasma cells, producing specific antitumor antibodies; (vi) Additionally, *Salmonella* is able to suppress tumor growth inducing inflammasome, via activation of interleukin-1*β* (IL-1*β*) and TNF-*α;* (vii) examined an attenuated strain of *Salmonella* engineered to express a truncated human interleukin-2 (IL2) protein called SalpIL2 (*Salmonella* encoding IL-2 (SalpIL2). In murine models, a single oral dose of SalpIL2 reduced primary tumor volume and the number of metastatic lesions as a result of increased tumor-targeted NK-cell activity. The attenuated strain of *S. typhimurium* labeled by green fluorescent protein (GFP) is also auxotrophic for leucine and arginine (termed *S. typhimurium* A1).

These attenuated strains have the potential for selective amplification and to be selectively infected within tumors. Hence, these bacteria are able to be used as a vehicle to target human tumors in vivo. Furthermore, they could express effector genes that encode the therapeutic proteins. It was indicated in a study that *Salmonella typhimurium* was attenuated by chromosomal deletion of the *purI* and *msbB* genes to raise safety and decrease the cytotoxic effect [97]. The *PurI* gene was deleted to increase dependence and the requirement for an external source of adenine [98]. The deletion of the *msbB* gene was associated with reducing toxicity by decreasing the stimulation of proinflammatory cytokines (TNFα) and nitric oxide [106]. The genes that delete strains are genetically stable; they have no antibiotic resistance markers, can decrease the tumor growth, prolong survival, and increase the number of bacteria in the tumor. The attenuated *Salmonella typhimurium* shows the anticancer activity against various cell lines of breast cancer, including MDA-MB-435, MDA-MB-361, MDA-MB-231, 4T1, Caco2, RKO, and MCF7 [99]. The attenuated strains of *S. typhimurium*, which are labeled by green fluorescent protein (GFP), are also auxotrophic for leucine and arginine, termed a *S. typhimurium A1*. This amino acid auxotrophic bacterium has unique antitumor efficacy, which can selectively grow in the viable tumor tissue and throughout the tumor including the viable malignant tissue. In addition, intravenous or intratumoral injection indicated that there were not any obvious side effects on the host. To increase the tumor-targeting capability of A1 the strain was re-isolated, by a study, after infection of the human colon tumor growing in nude mice, termed A1-R; with this passage through the HT-29 tumor, the ability of A1 was increased to adhere to and invade tumor cells [18]. The result of this study demonstrated that A1-R bacteria can be targeted, colonized, and replicated in the human MARY-X breast tumors growing s.c. in nude mice, as observed by fluorescence imaging on the second day after intravenous injection. The intravenous injection highly effected the targeting of the tumor and regressed human breast cancer. Moreover, the treated animals were completely cured and survived [18] (Table 4) (Figure 1).

### 4.2. Listeria Monocytogenes

**Figure 2:**
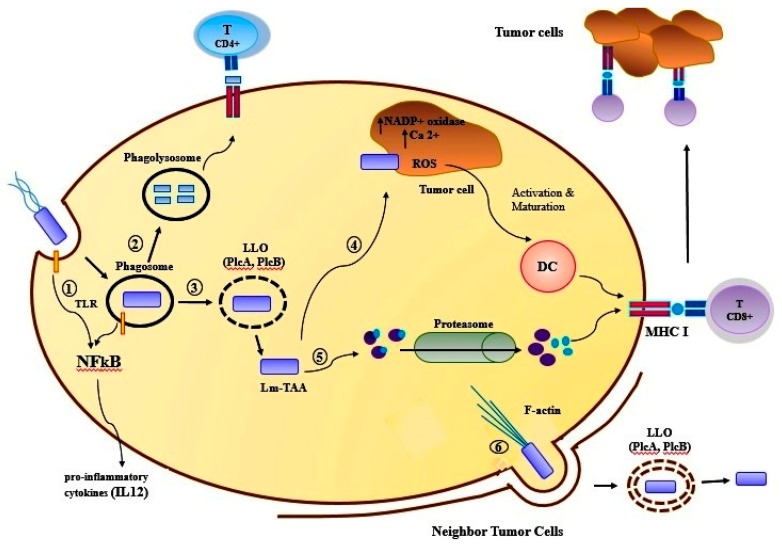
Innate and adaptive immune responses to recombinant *Listeria***.** (i) *L. monocytogenes* (Lm) is internalized by antigen-presenting cells into phagosomes. During entry, Lm is sensed by Toll-like receptors leading to the activation of NFκ-B and synthesis of proinflammatory genes. (ii) Lysosome fuses with phagosome to form a phagolysosome, where Lm can be killed and lysis, then, leading to loading of its antigens onto MHC class II for activation of CD4+ helper T cells. (iii) Lm can express the pore-forming toxin listeriolysin O (LLO) and two listerial phospholipases, PI-PLC and PC-PLC, to perforate phagosomes and gain entry into the cytosol. (iv) The *Listeria*-based vaccine has a dual mode of action, first, it is able to kill tumor cell via activation of NADP+ oxidase and, secondly, raise the intracellular level of Ca 2+, both resulting in the production of high ROS levels. A high level of ROS in tumor cell is able to induce the activation and maturation of dendritic cells (DC) which caused activation of CD8+ cytotoxic T lymphocytes. (v) Lm antigens into the cytosol where they can be degraded by proteasomes and loaded onto MHC class I for activation of TAA-specific (tumor-associated antigens) CD8+ cytotoxic T lymphocytes. (vi) Additionally, Lm is able to cell-to-cell spread via the formation of actin tails. This process results in the formation of double-membraned vacuoles from which the bacteria rapidly free themselves by LLO and cooperative action of the two listerial phospholipases PI-PLC and PC-PLC.

*Listeria monocytogenes* is an intracellular pathogen, which can have direct access to the cytoplasm of APC by the hemolytic activity of listeriolysin O (LLO). The infection caused by this bacterium is a classic model to induce the response of protective cellular immunity. Listeriolysin O (529 acid amine) has a hemolytic activity, can perforate the phagosomal membrane, and then the bacterium can escape from the vacuole to the cytoplasm [107]. These bacteria are widely used as a vaccine vector and this vaccine is used for generating the immune responses against the breast cancer (4T1, MDA-MB-231) and other human tumors in phase I and II of clinical trial. Lm-LLO-E7 is a recombinant *Listeria monocytogenes* (rLm) that secretes E7 as a fusion protein in conjugation with the non-hemolytic listeriolysin O (LLO). E7 is a protein produced by human papillomavirus-16 (HPV-16), associated with cervical cancer cells [100]. The other form of rLm is called ADXS31-142, which expresses HER2/neu oncogene as a fusion protein to a non-hemolytic fragment of listeriolysin O (LLO) by the highly attenuated *Listeria* vector LmddA. This vector can spread on a cell-to-cell basis, but it does not have any selection markers for antibiotics [101,102]. The results of the in vitro and in vivo studies indicate the attenuated *Listeria monocytogenes* (LM) based vaccine that expressed listeriolysin O (LLO) and amino acid fragments 311 to 660 of TAA Mage-b, called LM-LLO-Mage-b _311-660_. This *Listeria*-based vaccine has a dual mode of action; first, it can kill the tumor cells via activation of NADP^+^ oxidase and raise the intracellular level of Ca^2+^, both resulting in the production of high ROS levels, in vitro. The second action is the ability to induce strong CTL responses and depletion of TCD8^+^, which has resulted in killing the breast tumor cells in mouse breast tumor model 4T1 [108] (Table 4) (Figure 2).

### 4.3. Bifidobacterium Longum

*Bifidobacterium longum* belongs to the genus *Bifidobacterium* species that are part of the normal human microflora and exert probiotic effects in humans [109]. This bacterium can selectively germinate and grow in the hypoxic regions of solid tumors after intravenous injection [110]. The genetically engineered *B. longum,* used for enzyme-prodrug therapy, involves a fusion of cytosine deaminase and 5-fluorocytosine (5FC). The cellular toxicities of 5FC are due to the deamination via cytosine deaminase to give 5 fluorouracil (5FU) [111]. Furthermore, a plasmid, i.e., pBLES100-S-eCD, was constructed to adapt enzyme-prodrug therapy, which used *B. longum* as a tumor-specific gene delivery system. This construction includes the HU gene promoter and the cytosine deaminase gene of *E. coli*. The HU gene is able to encode a histon-like DNA binding protein highly expressed in *B. longum* [112]. The result of these studies shows that the transfected *B. longum*, which produces cytosine deaminase in the hypoxic tumor therapy can be an effective enzyme-prodrug therapy for the systemic administration.

### 4.4. Lactobacillus

*Lactobacillus plantarum* is a gram-positive lactic acid bacterium and a member of the genus *Lactobacillus*, which is commonly present in many fermented food products [113]. It has preventive and antitumor effects via immunomodulatory mechanisms on multiple solid tumors, as well as breast cancer. The chemopreventive ability of *L. plantarum* LS/07 (PRO), i.e., the prebiotic oligofructose-enriched inulin (PRE) and PRO-PRE combination were assessed in a rat model with breast cancer through oral administration. The results demonstrated that PRO, PRE, and PRO-PRE combination are significantly able to suppress the tumor frequency, raise the TCD4+ in tumor tissue, and decrease the serum level of TNFα. Furthermore, PRO and PRO-PRE can reduce the level of TCD8+ in blood and their increase in the tumor tissue [114]. CD8+ (cytotoxic) T cells can kill the tumor cells directly as well as breast tumor cells with higher levels of TCD8+ cells infiltration related to better survival rates among patients [115]. The T helper cells (Th cells), also known as CD4+ cells, show a heterogenous class of T-cells and help the response of TCD8+, particularly in the initial stages of tumor progression [116]. Different studies have demonstrated that *L. plantarum* strains are able to inhibit the growth and proliferation through G0/G1 phase cell-cycle arrest in breast cancer cell lines including MCF-7, MDA-MB-231, and 4T1 [103,117,118,119,120].

*Lactobacillus casei* CRL 431 is another member of genus *Lactobacillus*, which can suppress the growth of the tumor cells. The result of a study indicates that milk fermented by *L. casei* can decrease or inhibit the tumor growth in a murine breast cancer model. The result showed the ability to modulate the immune response by reducing the infiltration of macrophages in the tumor and also increase the level of T CD8+ and TCD4+ [121].

Cancer stem cells (CSC) present in solid tumors and hematological malignancies have direct and indirect roles in the induction of metastasis [122]. CSCs can be used for molecular targeting with therapeutic potential for the effective cancer therapy [123]. Several transcription factors and signaling pathways participate in supporting the CSCs, such as hypoxia-inducible factor (HIF), which facilitates the transcriptional responses in both normal and cancer tissues under hypoxia conditions, and also regulates the tumorigenic potential of glioma stem cells in hypoxic conditions. Furthermore, they can induce particular signaling pathways and transcription factors, including Notch and Oct4, which are associated with the self-restoration of stem cells and multipotency [105,124]. *Lactobacillus rhamnosus* belongs to the genus *Lactobacillus*, which can downregulate the expression of HIF-1α in breast cancer cell lines such as MDA-MB-231. HIF-1α signaling in the stem cells of mouse lymphoma and human acute myeloid leukemia has a specific activity and effects on the responsible inhibitors for inhibiting the preferential eradication of CSCs in the mouse models. *L. rhamnosus* with modulation of HIF-1α signaling can be used as a therapeutic agent (Table 4) [105].

## 5. Conclusions

Although some bacteria or their products may be attempted to treat cancer, their clinical application has not been used so far. Recently, many studies and animal trials have demonstrated that bacteria are useful tools for treatment of cancer, as vaccines that can induce the immune system or as vectors that can transmit the antitumor therapeutic agents. Some of these substances have multifunctional activities, for example, having antimicrobial and antitumor features. Similar to other treatment methods, this type of treatment also has adverse uncontrollable side effects, including developing infections or leading to death, due to infections by pathogens. Furthermore, to enhance the bacterial peptide or protein half-life and improve the target delivery of breast cancer and other cancer cell line, as well as metastatic and multidrug-resistant cells, using genetic engineering. Different peptide modification and genetic engineering approaches have been used to enhance the bacterial compound including chemical modifications, D-amino acid substitution, cyclization, replacement of labile amino acids, etc. [125,126]. Additionally, tumor-targeting peptides (TTP) have been used to specifically target different types of cancer cells and tumor-specific receptors. Moreover, TTP could be used as an effective delivery vehicle for chemical agents to the cancer site specifically with minimal effect on the normal cells. Different types of TTP have been identified which have been used to distinguish tumor angiogenesis or as a tumor cell killer to inhibit tumor progression [127].

Nevertheless, numerous studies have used the attenuated or genetically modified species of bacteria to overcome the side effects, which are considered safe with less or no side effects. Bacteria can use antioncogenes or immunogenic antigens and modified antitumor agents, and their anticancer potential is enhanced in combination with therapeutic processes. Using bacteria and their products as tools for the treatment of breast cancer with selective toxicity is still new and further studies are required to prove the clinical significance of bacteria-based cancer therapy. Additionally, although there have been promising results, most studies on anticancer therapeutic compounds of bacteria, such as peptide and proteins, have been stopped in the in vitro stage and only a few studies have gone from in vitro by clinical trial to registration and use as medicines. Therefore, this approach in the field of anti-breast cancer treatment needs further in vivo research, as well as clinical trials.

## Figures and Tables

**Table 1:** The origin and biological activity of anticancer bacteriocins.

Bacteriocins	Source	Breast Cancer Cell Line	Other Human Cancer Cells/Cell Lines	Ref.
Bovicin HC5	*Streptococcus bovis* HC5	MCF-7	Liver, hepatocellular carcinoma (HepG2)	[44,45]
Colicin E1 and A	*Escherichia coli*	MCF7, ZR75, BT549, BT474, MDA-MB-231, SKBR3 and T47D	Osteosarcoma (HOS), fibrosarcoma (MRC5, HS913T), leiomyosarcoma (SKUT-1 cells), lung cancer (A-549, PC-14, RERF-LC-AI), ovarian carcinoma, colon cancer (HCT116)	[44,46]
Laterosporulin 10	*Brevibacillus* sp. strain SKDU10	MCF-7	cervical cancer (HeLa), embryonic kidney cancer (HEK293T), fibrosarcoma (HT1080), lung carcinoma (H1299)	[47]
Nisin A	*Lactococcus* *lactis*	MCF-7	Head and neck squamous cell carcinoma (UM-SCC-17B, UM-SCC-14A, HSC-3), colon cancer (LS180, SW48, HT29, Caco2), liver hepatocellular carcinoma (HepG2), acute T cell leukaemia (Jurkat)	[48]

**Table 2:** The origin and biological activity of anticancer peptides.

Protein/Peptide	Source	Breast Cancer Cell Line	Other Human Cancer Cells/Cell Lines	Ref
Ohmyungsamycins A and B	*Streptomyces* sp.	MDAMB231	diverse cancer cells, colon cancer (HCT116), lung cancer cells (A549), stomach cancer (SNU638), liver cancer (SKHEP1)	[50]
Azurin	*Pseudomonas aeruginosa* strains	MCF7, ZR-75-1, T47D, MDA-MB-157, MDD2, MDA-MB-231	Normal melanocytes (HFB4), liver cell line (HEPG2), colon cell line (HCT116), progressive pediatric CNS tumors	[51,52]
Pep27anal2	Streptococcus pneumoniae	MCF-7	leukemia cells (AML-2, HL-60, Jurkat), gastric cancer cells (SNU-601)	[53,54]
Entap	*Enterococus sp. strains*	MDA-MB-231	Gastric adenocarcinoma cells (AGS), uterine cervix adenocarcinoma cells (HeLa), prostate carcinoma (22Rv1), colorectal adenocarcinoma (HT-29)	[43,55,56]
Proximicins	Verrucosispora sp. MG-37 and AB-18-032	MCF 7	Human gastric adenocarcinoma (AGS), human hepatocellular carcinoma (HepG2)	[57]
Urukthapelstatin A	Mechercharimyces asporophorigenens YM11-542	MCF-7	Human lung cancers (A549, DMS114, NCIH460), ovarian cancers (OVCAR-3, OVCAR-4, OVCAR-5, OVCAR-8, SK-OV3), colon cancer (HCT-116)	[58]

**Table 3:** The origin and biological activity of anticancer toxin of bacteria.

Toxins	Source	Breast Cancer Cell Line	Other Human Cancer Cells/Cell Lines	Ref
Diphtheria toxin	*Corynebacterium diphtheria*	MCF 7	Adrenocortical carcinoma (H295R), glioblastomas (U118MG, U373MG, U87MG), cutaneous T cell lymphomas (CTCL), cervical adenocarcinoma (HeLa), colon cancer (SW480, SW620, HCT116, CaCo-2, and HT-29)	[66,67,68]
Botulinum neurotoxin type A	*Clostridium botulinum*	T47D, MDA-MB-231, MDA-MB-453	prostate cancer (PC-3, LNCaP), neuroblastoma (SH-SY5Y)	[69,70]
Exotoxin A	*Pseudomonas aeruginosa*	MCF-7, BT-20, CAMA-1, SKBR-3	pancreatic cancer (PaCa-2), melanomas (FEMX, Melmet-1, Melmet-5, Melmet-44, MelRM, MM200), head and neck squamous carcinomas, Burkitt’s lymphoma (Daudi, CA46), leukemias (EHEB, MEC1)	[71,72]
Exotoxin T	*Pseudomonas aeruginosa*	MDA-MB-231, EMT6, 4T1	murine-derived fibrosarcoma cell line (MCA-205), human melanoma (A375), human lung adenocarcinoma (Calu-3), murine lung carcinoma (LLC1), human ovarian adenocarcinoma (SK-OV-3), human cervical adenocarcinoma (HeLa)	[73]
Hyaluronidases (Hyals)	*Streptococcus pyogenes*	Hs578T, MDA-MB-231, MCF-7		[74]

**Table 4:** The origin and biological activity of bacteria as a carrier for cancer therapeutic agents.

Bacteria as a Carrier	Source	Breast Cancer Cell Line	Other Human Cancer Cells/Cell Lines	Clinical Phase	Ref
*Salmonella typhimurium* (VNP20009)	*Salmonella*	MDA-MB-435, MDA-MB-361, MDA-MB-231, 4T1, Caco2, RKO, and MCF7	Metastatic melanoma (MDA-MB-435, B16-F10), human glioblastoma (U87MG), human pancreatic cancer (ASPC-1), colon carcinoma (WiDr, CT26)	Phase I	[97,98,99]
Listeriolysin O (Lm-LLO-E7)	*Listeria monocytogenes*	4T1, MDA-MB-231	Cervical cancer, human acute monocytic leukemia cell line THP-1, human ovarian cancer (SKOV3-A2), human prostate cancer (LNCaP), and human colon cancer (Colo205)	Phase I/II	[100]
Listeriolysin O (ADXS31-142)	*Listeria monocytogenes*	EMT6-Luc	Prostate cancer, breast cancer, colorectal cancer, pancreatic cancers(EMT6-Luc, HLA-A2, NT-2 cell line)	Phase I/II	[101,102]
*Lactobacillus plantarum*	*Lactobacillus*	MCF-7, MDA-MB-231, 4T1	Colon cancer (Caco-2, BGC-823, HT-29)		[103,104]
*Lactobacillus rhamnosus*	*Lactobacillus*	MDA-MB-231	Colorectal cancer (HT-29), cervical adenocarcinoma (HeLa)		[105]
